# Prior knowledge guided active modules identification: an integrated multi-objective approach

**DOI:** 10.1186/s12918-017-0388-2

**Published:** 2017-03-14

**Authors:** Weiqi Chen, Jing Liu, Shan He

**Affiliations:** 1 School of Computer Science, University of Birmingham, Edgbaston, Birmingham, B15 2TT UK; 2 Key Laboratory of Intelligent Perception and Image Understanding of Ministry of Education, Xidian University, Xi’an, Shaanxi, 710071 People’s Republic of China

**Keywords:** Prior knowlege, Multi-objective evolutionary algorithm, Active module identification

## Abstract

**Background:**

Active module, defined as an area in biological network that shows striking changes in molecular activity or phenotypic signatures, is important to reveal dynamic and process-specific information that is correlated with cellular or disease states.

**Methods:**

A prior information guided active module identification approach is proposed to detect modules that are both active and enriched by prior knowledge. We formulate the active module identification problem as a multi-objective optimisation problem, which consists two conflicting objective functions of maximising the coverage of known biological pathways and the activity of the active module simultaneously. Network is constructed from protein-protein interaction database. A beta-uniform-mixture model is used to estimate the distribution of *p*-values and generate scores for activity measurement from microarray data. A multi-objective evolutionary algorithm is used to search for Pareto optimal solutions. We also incorporate a novel constraints based on algebraic connectivity to ensure the connectedness of the identified active modules.

**Results:**

Application of proposed algorithm on a small yeast molecular network shows that it can identify modules with high activities and with more cross-talk nodes between related functional groups. The Pareto solutions generated by the algorithm provides solutions with different trade-off between prior knowledge and novel information from data. The approach is then applied on microarray data from diclofenac-treated yeast cells to build network and identify modules to elucidate the molecular mechanisms of diclofenac toxicity and resistance. Gene ontology analysis is applied to the identified modules for biological interpretation.

**Conclusions:**

Integrating knowledge of functional groups into the identification of active module is an effective method and provides a flexible control of balance between pure data-driven method and prior information guidance.

## Background

With the development of high-throughput data collection technologies, vast amounts of omics data that cover different species and different levels of biological activities have accumulated exponentially. These varied omics data, including the genome sequencing data (genomics), genome-wide expression profiles (transcriptomics), and protein abundances data (proteomics), provide valuable information concerning the intrinsic mechanisms underlining biological processes. With the accumulation of large datasets, one of the most essential challenges for researchers is that how to properly interpret these data. Take gene expression data analysis as an example, methods have evolved from the simple single or multivariate statistical analysis, e.g., calculation of fold-change, identification of differential expressed genes, to integrated approaches that integrate prior knowledge and different datasets [1]. As a research field driven by those integrated approaches, network biology has gained popularity recently years.

Network biology offers a highly abstract model of networks to characterize various levels of biological systems and provides insights into those system by taking advantages of network theory [2, 3]. Although currently it’s not able to fully capture the diversity and dynamics of complex biological system [4], it is still one of the most promising and fast developing research area in modern biology. Many studies have been performed on the construction of networks from biological systems and the structural and functional features that may respond to related biological information. Network construction methods are varied from calculating pair-wise correlation coefficient of expression data (correlation network [5]), filtering from existing interaction database (protein-protein interaction network [6–9]), or integrated approaches based on both expression data and metabolic models (tissue specific metabolic network [10]). Structural features includes degree distribution, clustering coefficient, scale-free property [11], modularity [12] and network robustness [13]. One of the most studied features is modular structure.

Modular structure is one of the essential characteristics that reveal information about the relationship and interaction among components in the network. In biological networks, modules are considered as the functional units of cellular process and organization [14]. Varied definitions of module have been proposed and numerous methods have been developed to identify those modules [15, 16], all aiming to reveal essential biological mechanisms [17, 18]. Among them, active module detection is a successfully applied integrative approach. Active module is a densely connected area in network that shows striking changes in molecular activity or phenotypic signatures, which is often associated with a given cellular response. Active module is expected to reveal dynamic and process-specific information that is correlated with cellular or disease states.

A typical active module detection algorithm takes gene expression data, calculates statistical values indicating differential expression level, and searches in corresponding network to identify modules inside which gene activity changes significantly. The jActiveModule [19] method proposed by Ideker in 2002 is considered as the first to formulate active module detection into an optimization problem. It uses the standard normal inverse of single gene’s *p*-value to measure the activity of one gene, aggregates the node scores for a given module with adjustment and background correction, and finally searches for high-scoring modules via simulated annealing. Many following methods adopt this framework of significant-area-search method. One representative research for identifying condition-responsive protein-protein interaction module used edge-based scoring method [6]. There are also formulations that combine both node and edge score [7, 9]. As the problem of finding the maximal-scoring connected subgraph is NP-hard (non-deterministic polynomial-time hard) [19], heuristic algorithms are broadly used, e.g. simulated annealing [19], greedy search [20], and evolutionary algorithm [8, 21]. Exact approaches via integer linear programming are also developed [22].

In this paper we propose a novel multi-objective active module identification algorithm. We first formulate the active module identification problem as a multi-objective problem, which not only maximises the activity score as defined by Dittrich and Klau [22] but also maximises the prior knowledge contained in the active module. The intuition behind this multi-objective formulation is to use prior knowledge to guide the search of novel information from data, i.e., active modules. The Pareto solutions from this multi-objective optimisation problem are then the optimal trade-off between known knowledge and novel information.

In order to solve this multi-objectie problem, we proposed a modified multi-objective evolutionary algorithm. One of the important details omitted in many papers of active module identification is how to ensure the connectedness of the solutions. Without this connectedness constraint, the optimal solution is trivial, i.e., the top genes with largest node scores. In order to ensure the connectedness of the identified active modules, we design a novel constraints based on algebraic connectivity. The algorithm is applied to a small molecular interaction network that was used by Ideker [19] and then applied to a large Protein-Protein Interaction (PPI) network constructed from microarray data on drug toxicity and resistance.

## Methods

### Problem formulation

The network *G* is represented as *G*=(*V,E*) with *p*_*v*_∈(0,1) for *v*∈*V* where *V* is the set of nodes, *E* the set of edges, and *p*_*v*_ the assigned *p*-value from differential expression analysis for each node *v*. In the proposed algorithm there are two objectives and one constraint for a given module *A*:

- Active module score *S*_*A*_ indicating significant changes in gene expression for a given module, to be maximized during search.
- KEGG (Kyoto Encyclopedia of Genes and Genomes) pathway coverage score *R*_*A*_ for the number of covered metabolic pathway by genes in module, to be maximized.
- Algebraic connectivity to check whether a given subgraph is connected or not, used as a constraint to ensure connectedness.

#### Active module score

Microarray analysis studies showed that expression data can be effectively estimated by many mixture-model methods that divide genes into two or more groups, one group contains genes that are differentially expressed, and other(s) not differentially expressed [1]. Among those many methods, Pounds and Morris proposed a beta-uniform mixture (BUM) model that very accurately describes the distribution of a large set of *p*-values produced from an microarray experiment [23]. The BUM model considers the distribution of *p*-values as a mixture of a special case of beta distribution (*b*=1) and a uniform(0, 1) distribution, with a mixture parameter *λ*. The *p*-values under the null hypothesis are assumed to have a uniform distribution. Under the alternative hypothesis the distribution of *p*-values will have a high density for small *p*-values and can be described by *B*(*a*,1).

A general beta distribution *B*(*a,b*) is given by

1 $$ f(x)= \frac{\Gamma(a+b)}{\Gamma(a)\Gamma(b)}x^{a-1}(1-x)^{b-1}  $$

where *Γ*(.) denotes the gamma function. As *Γ*(1)=1, the probability density function of BUM model is then reduced to

2 $$ f(x|a, \lambda)=\lambda+(1-\lambda)ax^{a-1}  $$

for 0<*x*≤1, 0<*λ*<1 and 0<*a*<1. Given a set of *p*-values the two parameters of BUM distribution *λ* and *a* can be calculated by maximum likelihood estimation.

Following the idea of Dittrich and Klau [22] to decompose signal component from background noise, an additive score to measure the significance of gene’s differential expression is calculated as

3 $$\begin{array}{@{}rcl@{}} S^{FDR}(x) &=& \log \frac{B(a, 1)(x)}{B(a, 1)(\tau)}\\ &=& \log \frac{ax^{a-1}}{a\tau^{a-1}}\\ &=& (a-1)(\log x - \log \tau) \end{array} $$

where *τ* is a threshold to determine the significance of a *p*-value. In order to control the estimated upper bound of the false discovery rate (FDR) introduced by Benjamini and Hochberg [24], *τ* could then be selected to ensure that *FDR*≤*α* for some predefined *α* using the following equation

4 $$ \tau = \left(\frac{\hat\pi - \alpha\lambda}{\alpha(1-\lambda)}\right)^{\frac{1}{(a-1)}}  $$

where $\hat \pi = \lambda + (1-\lambda)a$, meaning the maximum proportion of the set of *p*-values that could arise from the null hypothesis.

After assigning score to each of the genes, the overall score for a given module *A* is then the summation of all genes’ scores in it, given by

5 $$ S_{A} = \sum_{x\in A}S^{FDR}(x)  $$

#### KEGG pathway coverage

KEGG is an integrated database of high level functions and utilities of biological systems [25]. KEGG PATHWAY is a collection of manually drawn pathway maps representing the knowledge on the molecular interaction and reaction networks. Mapping of pathway information mainly relies on molecular datasets, especially large-scale datasets such as genomics, transcriptomics, proteomics, and metabolomics. Genes involved in the same KEGG pathway are considered as functionally related to each other. In the experiment KEGG pathway coverage score *R*_*A*_ is formulated as the second objective to measure the enrichment of functional groups in a given module *A*.

The KEGG pathway information is retrieved from the KEGG REST-style entry for *Saccharomyces cerevisiae* (yeast) [26]. Each entry of the mapping data records one gene and its corresponding pathway. The records are then split into different groups labeled by the pathways. For the *i*-th pathway, *V*_*i*_ stands for the set of genes it contains. Given a module *A* with *V*_*A*_ as the set of genes, its KEGG pathway cover rate *R*_*i*_ over the *i*-th pathway is calculated as

6 $$ R_{i} = \frac{|V_{i} \cap V_{A}|}{|V_{i}|}  $$

meaning the percentage this pathway is covered by given module. The cover rate *R*_*i*_ is then compared with a threshold *R*_*ratio*_ to determine whether this pathway can be considered as enriched in the given module. The threshold shall be selected carefully. A too high value of *R*_*ratio*_ leads to a tiny group of connected pathways genes with positive active module score as the search could not expand to other area under such stringent condition. On the contrary, a very low *R*_*ratio*_ could not reflect the meaning for the second objective. In practice, *R*_*ratio*_ is set to a series of values for preliminary experiment. The results are analyzed and compared to decide a suitable value. The total enriched pathway count *R*_*A*_ is given by

7 $$ R_{A} = |\{R_{i}|R_{i} > R_{ratio}\}|, i\in P  $$

where *P* stands for total number of pathways.

#### Algebraic connectivity

The algebraic connectivity of a graph *G*, denoted as *α*(*G*), is the second-smallest eigenvalue of the Laplacian matrix of *G*. It serves as a good parameter to measure how well a graph is connected. *α*(*G*) is greater than zero if and only if *G* is a connected graph.

The Laplacian matrix *L* of a simple graph *G* is calculated as

8 $$ L=D-A  $$

where *D* is the degree matrix and *A* the adjacency matrix. The eigenvector *ν* of the square matrix *L* is the non-zero vector that satisfies

9 $$ L\nu = \lambda \nu  $$

*λ* is a scalar known as the eigenvalue associated with the eigenvector *ν*. Algebraic connectivity *α*(*G*) is the second smallest eigenvalue of the Laplacian matrix *L*.

### Multi-objective optimization algorithm

In order to perform multi-objective optimization to maximize both active module score and KEGG pathway coverage score simultaneously, a multi-objective evolutionary algorithm modified from NSGA-II (non-dominated sorting genetic algorithm II, see [27]) is applied as search strategy for module detection.

#### Solution representation

A solution is represented as a binary vector of length |*V*|, where |*V*| is the size of network, i.e. total number of nodes. Adding or deleting a node is performed through simply flip one bit of the vector at corresponding site.

#### Fitness function

Active module score *S*_*A*_ and KEGG coverage score *R*_*A*_ are used as two objectives. As the implementation of the algorithm is aimed at minimization both objectives, scores calculated from above equations would be given an extra negative sign.

#### Initialization

The search starts by randomly initializing a group of small cores in network. Nodes with high *S* ^*FDR*^(*x*) scores are selected as seeds of potential modules to begin with. Number of seed nodes is decided by the population parameter for evolutionary algorithm. For instance, if population is set to 50, nodes with top 50 *S* ^*FDR*^(*x*) scores are selected as seeds. In the case when the population size exceeds network size, every node will be selected as a seed. In initialization stage, neighboring nodes of a seed with positive scores are added to the module in which the seed represents.

#### Parent selection

Binary tournament selection is applied for selecting parents to reproduce. In some cases when the population converges too fast, this step is skipped to decrease selection pressure, thus the whole population would be used for reproduction.

#### Reproduction

Single point crossover is applied to selected parents. Mutation is performed by adding neighboring nodes with positive *S* ^*FDR*^(*x*) score or in a pathway into current module each time. Offspring generated is added to parental population to form a combined population with twice the size, waiting to be sorted and selected.

#### Clearing procedure

An extra clearing procedure is applied after reproduction and before non-dominated sorting. The step is introduced because in practise the algorithm tends to generate a number of replicated solutions when converging towards global optima. However, considering the natural property of our optimization problem, it is reasonable to obtain multiple optima, both those global on the non-dominated Pareto front and those dominated local optima, each representing the most significantly changed modules and modules that do not change that significantly, but still worth looking into. This procedure, inspired and simplified from Petrowski [28], detects replicated solution groups, preserves one copy, and resets all other individuals as infeasible solutions which will soon be eliminated after soring and replacement.

#### Sorting and replacement

The algorithm uses fast non-dominated soring and crowding distance assignment as detailed in Ref [27] to generate new population from the combined population efficiently and preserve solution diversity.

#### Constraint handling

To ensure the connectivity of detected module after crossover, algebraic connectivity *α*(*G*) is used as a constraint. Solution with non-positive algebraic connectivity violates the constraint, indicating itself a disconnected subgraph and thus an infeasible solution. Replicated solutions are also marked infeasible in the clearing procedure. Infeasible solutions are dominated by all feasible solutions.

### Network construction

#### Network 1: a small molecular interaction network on galactose utilization pathway

A small molecular interaction network once used by Ideker [19] is used as a test network. The molecular interaction networks visualization software Cytoscape [29] provides jActiveModule as a plugin to find expression activated modules. The tutorial in Cytoscape App Store [30] provides samples data consists of a network file as a model of the galactose utilization pathway in yeast and a companion expression file contains *p*-values to describe the significance of each observed change in expression. *p*-values under condition labeled as *GAL80R* are extracted and overlaid to network file, resulting in a network with 330 genes.

#### Network 2: yeast drug reaction network constructed from differential analysis and interactome mapping

Gene expression data on yeast’s reaction to diclofenac is downloaded from GEO (NCBI Gene Expression Omnibus) database [31]. Diclofenac is a widely used analgesic drug that can cause serious adverse drug reactions [32]. Yeast is used as model eukaryote to capture the cellular changes under the treatment of diclofenac. The data provides the microarray expression for diclofenac-treated yeast cells and control cells, each with 5 replicates. Differential expression analysis between diclofenac-treated group and control group is performed using the on-line tool GEO2R [33], with *p*-value adjustment set to Benjamini and Hochberg false discovery rate control. After deleting genes with adjusted *p*-value larger than 0.05, a set of differentially expressed genes is generated for interactome mapping.

Protein-protein interaction data is download from BioGRID [34], an integrated and up-to-date public database that archives and disseminates genetic and protein interaction data from model organisms and humans. To be specific, the downloaded file is BIOGRID-ORGANISM-LATEST.tab2.zip that separates interactions into distinct files based on Organism and was released on June 30, 2016. File for interactions of *Saccharomyces cerevisiae* is extracted for use. As the whole interaction data contains tens of thousands of proteins and millions of interaction records, a considerable amount of proteins have no corresponding records in given expression data or show no differential expression. Those proteins shall be excluded from the final network in order to avoid the waste of both computational resource and analysis attention. According to the filtering method applied by Muraro and Simmons [8], interactions containing at least one differentially expressed gene are selected as an attempt to include indirect interactions. The resulting network concerning yeast cellular reaction to diclofenac consists of 1803 nodes and 3356 edges.

## Result and Discussion

### Analysis of network 1

**Fig. 1:**
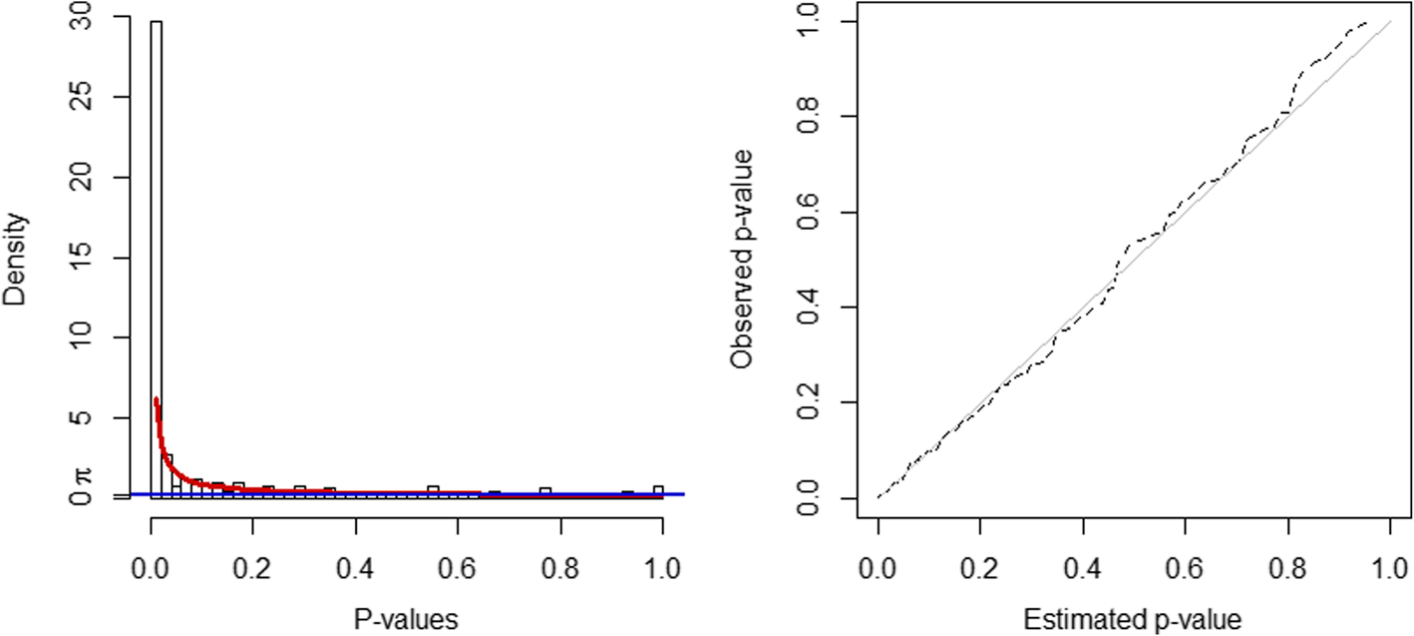
BUM model estimation on *p*-values in network 1. *Left* figure is a histogram of *p*-values with fitted beta-uniform-mixture model distribution. *Blue line* indicates the uniformly distributed noises and *red line* the signals as beta distribution *B*(*a*,1). *Right* figure is a Q-Q plot of the fitted distribution versus the empirical *p*-values for network 1

To estimate distribution for *p*-values, the parameters of BUM model *a* and *λ* are estimated by R package BioNet [35]. Figure 1 shows the fitted model. As the majority of genes in yeast network have a very significant *p*-value, threshold *τ* is calculated at an extremely stringent FDR level as an attempt to control the size of detected module. Parameter details are shown in Table 1.

**Table 1:** Parameters for experiment networks

Parameters	Network 1	Network 2
nodes	330	1803
edges	359	3356
*a*	0.113	0.280
*λ*	9.07×10^−2^	0.168
*α* (FDR)	1×10^−4^	1×10^−4^
*τ*	1.76×10^−4^	7.71×10^−6^
*R*_*ratio*_	0.6	0.8

**Fig. 2:**
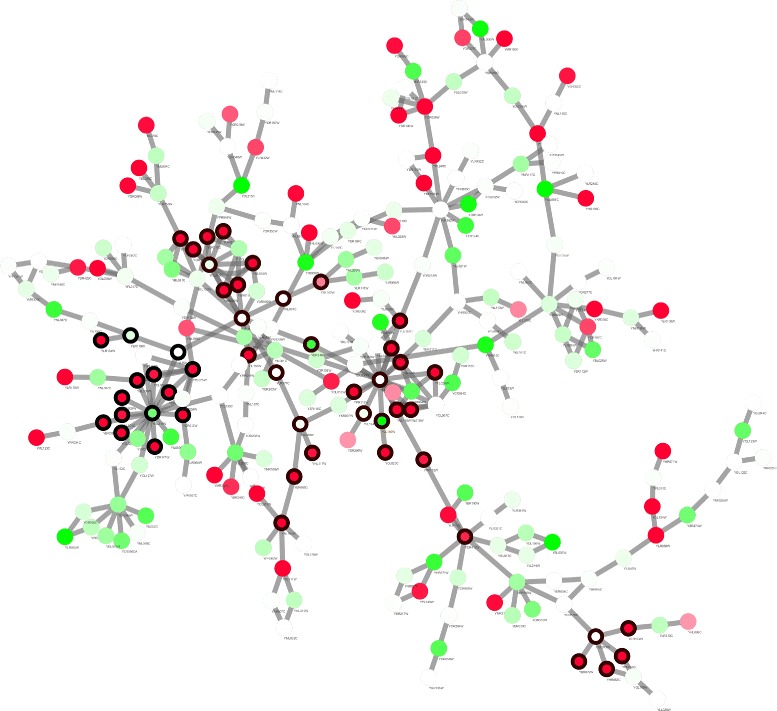
Network 1 with active modules detected by jActiveModule. Each node denotes for one gene. *Node color* is a continuous mapping of the *p*-value generated from differential expression analysis. *Red color* indicates a significant change with small *p*-value and green color means no significant difference. The point where color will change between *red* and *green* is set to the threshold *τ*=1.76×10^−4^ that is used as a parameter for the proposed algorithm. Color of nodes near the changing point is white. Modules identified by jActiveModule are highlighted with *black node* border. Modules may overlap with each other

As a benchmark, the jActiveModule method is applied to the network via Cytoscape plugin, generating 5 active modules by default. Figure 2 gives a visualization of the network by Cytoscape, with detected active modules mapped on it. To understand the biological function of modules, gene ontology (GO) annotation for biological process is applied to modules by enrichment analysis tools provided on Gene Ontology Consortium [36]. The tool only asks for a submission of gene list, GO type (biological process, molecular function, cellular component) and species. The results is shown in Table 2. Among the 5 modules, 3 modules are enriched in the GO term galactose catabolic process via UDP-galactose with *p*-values from 4.85×10^−05^ to 3.42×10^−04^. Other 2 modules are too tiny to have accurate explanation.

**Table 2:** Gene ontology results of modules detected by jActiveModule in network 1

Module	Size	*S*_*A*_	*R*_*A*_	Typical GO terms	*p*-value
1	26	250.39	1	galactose catabolic process via UDP-galactose	3.42×10^−04^
				glycolytic fermentation to ethanol	2.72×10^−03^
				amino acid catabolic process to alcohol via Ehrlich pathway	1.25×10^−02^
2	5	58.21	0	response to heat	2.16×10^−03^
3	16	270.79	2	galactose catabolic process via UDP-galactose	4.85×10^−05^
4	18	169.89	2	galactose catabolic process via UDP-galactose	1.15×10^−04^
				cellular carbohydrate metabolic process	3.27×10^−02^
5	4	37.05	0	None	Not available

*S*_*A*_ and *R*_*A*_ are the objective functions of active module score and KEGG pathway coverage score, respectively. The values are calculated by the proposed objective functions using the same parameters setting as the proposed algorithm. *τ*=1.76×10^−4^ and *R*_*ratio*_=0.6

The proposed algorithm is applied to network 1 with threshold *R*_*ratio*_=0.6 for KEGG pathway coverage score, resulting in a set of 13 Pareto solutions. As a feature for multi-objective optimization, all the modules in the same Pareto front are equally good. No one out performs another. In order to show the difference of those modules in trade-offs between two objectives, we selected 3 modules from the 13 Pareto solutions:

- Module 1: the extreme point on the Pareto front with maximum active module score *S*_*A*_=393.41.
- Module 2: at the knee point of the Pareto front, which represents the optimal trade-off between active score (*S*_*A*_=268.96) and KEGG pathway coverage score (*R*_*A*_=19)
- Module 3: the extreme point on Pareto front with maximum KEGG pathway coverage *R*_*A*_=25.

**Fig. 3:**
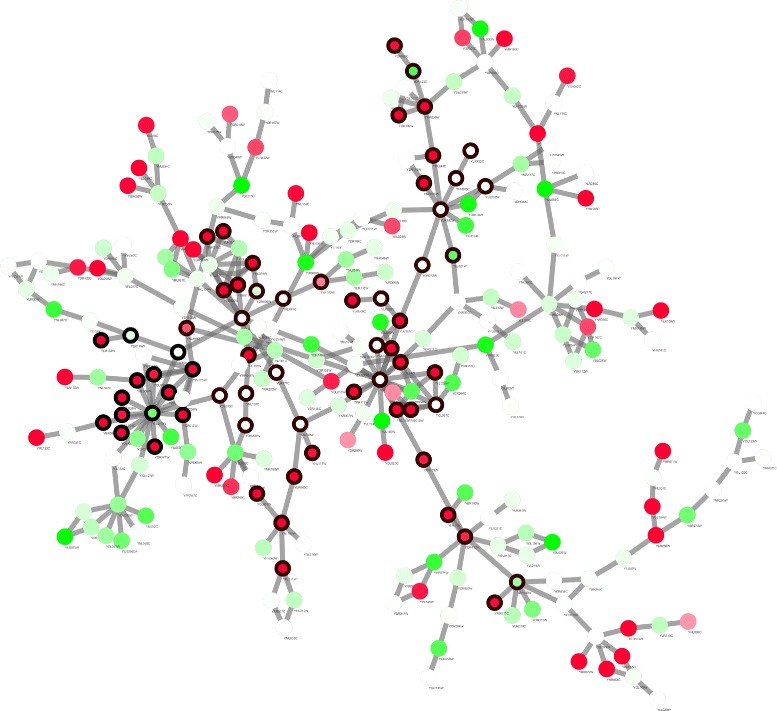
Visualization of Module 1 with maximized active score *S*_*A*_ detected by the proposed algorithm in network 1. *Node color* and *border* are set the same as Fig. 2. Module contains the majority of *red* nodes that are connected densely, indicating high activity. Notice that compared to small separated modules identified by jActiveModule shown in Fig. 2, this module tends to connect small areas of *red* nods by including linkage nodes with *white* or *light green* color. Although these intermediate nodes shows only modest changes in expression, they serve as bridges for cross-talk between functional groups, or as transcription factors that regulate other genes

**Fig. 4:**
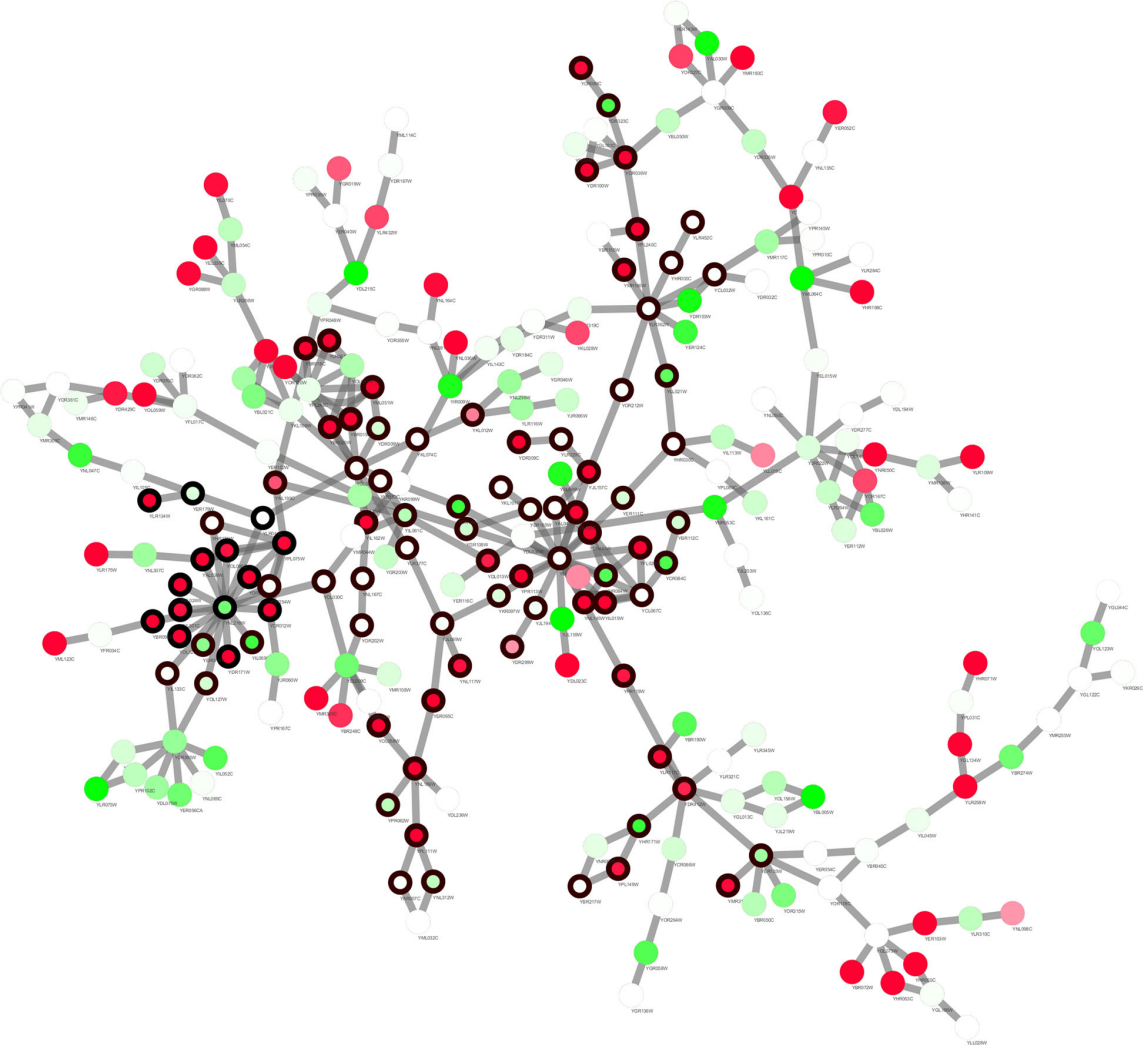
Visualization of Module 2 which is the knee point of the Pareto front with optimal trade-off between *S*_*A*_ and *R*_*A*_ detected by the proposed algorithm in network 1. *Node color* and *border* are set the same as Fig. 2. Compared to Fig. 3, this module expands broader as *R*_*A*_ gets higher

GO analysis for biological process is performed on the three modules. The results together with the objective function values are tabulated in Table 3. We also visualize Modules 1 and 2 in Figs. 3 and 4, respectively.

**Table 3:** Gene ontology results of 3 modules on Pareto front detected by the proposed algorithm in network 1

Module	Size	*S*_*A*_	*R*_*A*_	Typical GO terms	*p*-value
1	65	393.41	9	galactose catabolic process via UDP-galactose	5.15×10^−03^
				negative regulation of mating-type specific transcription from RNA polymerase II promoter	1.21×10^−02^
				glycolytic fermentation to ethanol	4.05×10^−02^
				pheromone-dependent signal transduction involved in conjugation with cellular fusion	6.39×10^−03^
				cellular carbohydrate metabolic process	4.16×10^−02^
2	92	268.96	19	negative regulation of mating-type specific transcription from RNA polymerase II promoter	4.67×10^−04^
				galactose catabolic process via UDP-galactose	1.63×10^−02^
				regulation of transcription during mitosis	7.19×10^−03^
				gluconeogenesis	1.84×10^−04^
				glycolytic process	2.87×10^−02^
				pyruvate metabolic process	4.20×10^−02^
				response to pheromone involved in conjugation with cellular fusion	3.93×10^−06^
3	126	181.3	25	negative regulation of mating-type specific transcription from RNA polymerase II promoter	1.80×10^−03^
				galactose catabolic process via UDP-galactose	4.48×10^−02^
				C-terminal protein lipidation	1.62×10^−02^
				gluconeogenesis	1.36×10^−03^
				ADP metabolic process	2.47×10^−04^
				pyruvate metabolic process	7.73×10^−05^
				response to pheromone involved in conjugation with cellular fusion	1.47×10^−02^
				ribonucleoprotein complex assembly	5.31×10^−03^

Module 1 is the extreme point with maximized active score *S*_*A*_. Module 2 is a balanced solution between *S*_*A*_ and *R*_*A*_. Module 3 is the other extreme point with maximized pathway coverage score *R*_*A*_

By comparing the results in Table 3 with those in Table 2, we found that Module 1 identified by the proposed algorithm have better active module score (*S*_*A*_) and KEGG pathway coverage score (*R*_*A*_) than all the modules found by jActiveModule algorithm. Such results indicate that by incorporating the prior knowledge, we can guide the algorithm to search areas in the network with more significant activity.

From these two figures and Table 3, we found that compared with jActiveModule that searches for small and separated modules, the proposed algorithm tends to identify a large connected subgraph. Even for Module 1 where the active module score is maximised, because of the integration of the prior knowledge, highly active areas are more likely to be connected together by intermediate nodes that might not be significantly differential expressed, but serve as a bridge for cross-talk between neighboring functional areas.

By visualisation of those Pareto solutions (figures not shown), we found that as the solution on Pareto front moves from maximum active score to maximum pathway coverage score, such intermediate nodes appear with higher frequency. We also found that, as *R*_*A*_ gets higher, detected module expands from a small core area with high activity to a broad area with more varied functional groups while still keeping overall activity. This result indicates that by using prior knowledge, we are able to reveal underlying mechanisms that link different activities in the network.

While all the three modules are significantly enriched in the GO term “galactose catabolic process via UDP-galactose” (corresponding *p*-value 5.15×10^−03^, 1.63×10^−02^ and 4.48×10^−02^, respectively), annotations for Module 1 (the extreme point with maximum activity score *S*_*A*_) are more densely related with galactose metabolic process. On the other hand, for Module 3 with maximum KEGG pathway coverage score *R*_*A*_, core annotations remain the same while additional annotations concerning essential biological processes increases. However, it is worth noting that, all the additional annotations can be reasonably related to the cellular response to disturbance in galactose utilization pathway.

The most interesting module is Module 2, which represents the optimal trade-off between prior knowledge and novel information from data. It is worth noting from Tables 3 and 2 that, even it is a knee point solution, Module 2 has a slightly worse *S*_*A*_ but much higher *R*_*A*_ than all the modules identified by JActiveModule. We can also observe from Table 3 that, module 2 has a range of slightly broader annotations concerning metabolic process of galactose, pyruvate and gluconeogenesis, which are highly relevant to galactose untilization pathways [37].

### Analysis of network 2

**Fig. 5:**
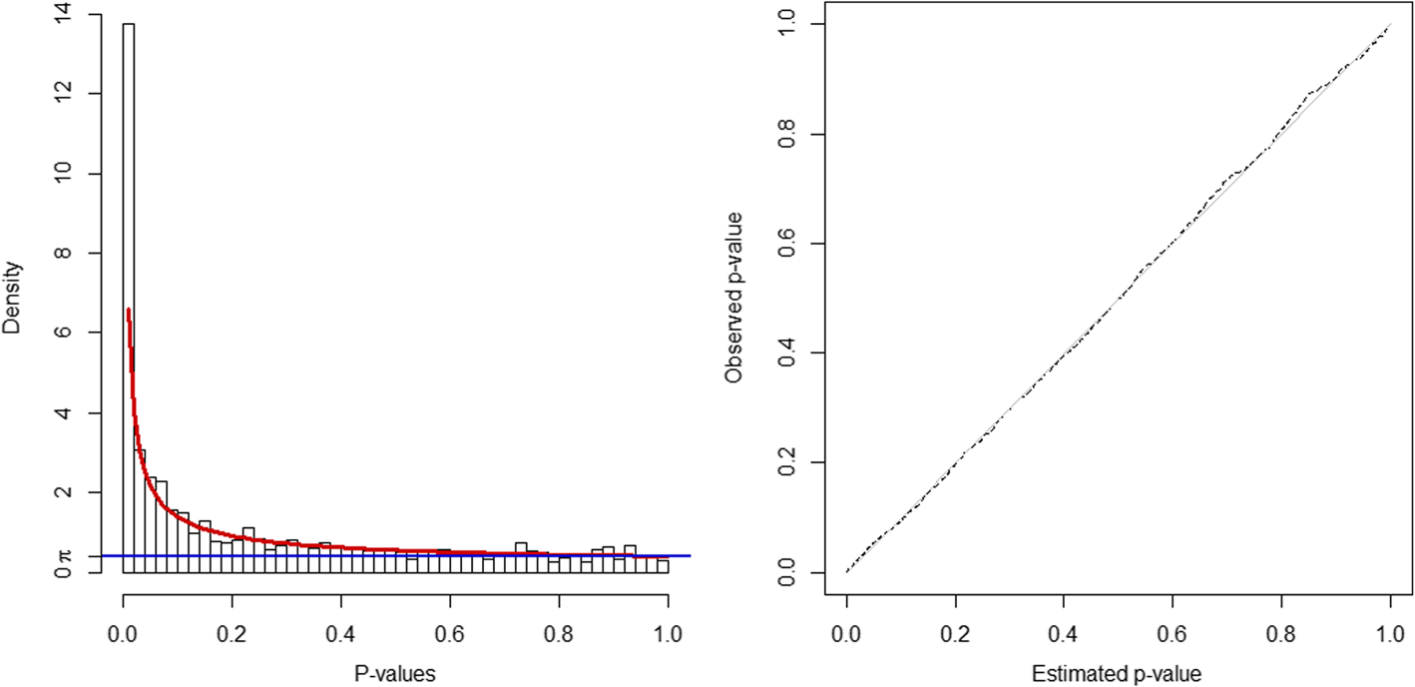
BUM model estimation on network 2. Histogram of *p*-values with fitted BUM model and a Q-Q plot of estimated and empirical distribution of *p*-values for network 2. As the network size increases, estimation becomes more accurate

Parameters of BUM model *a* and *λ* to fit *p*-value distribution are estimated as shown in Fig. 5. Threshold *τ* is calculated at given FDR level. See Table 1 details of parameters.

**Fig. 6:**
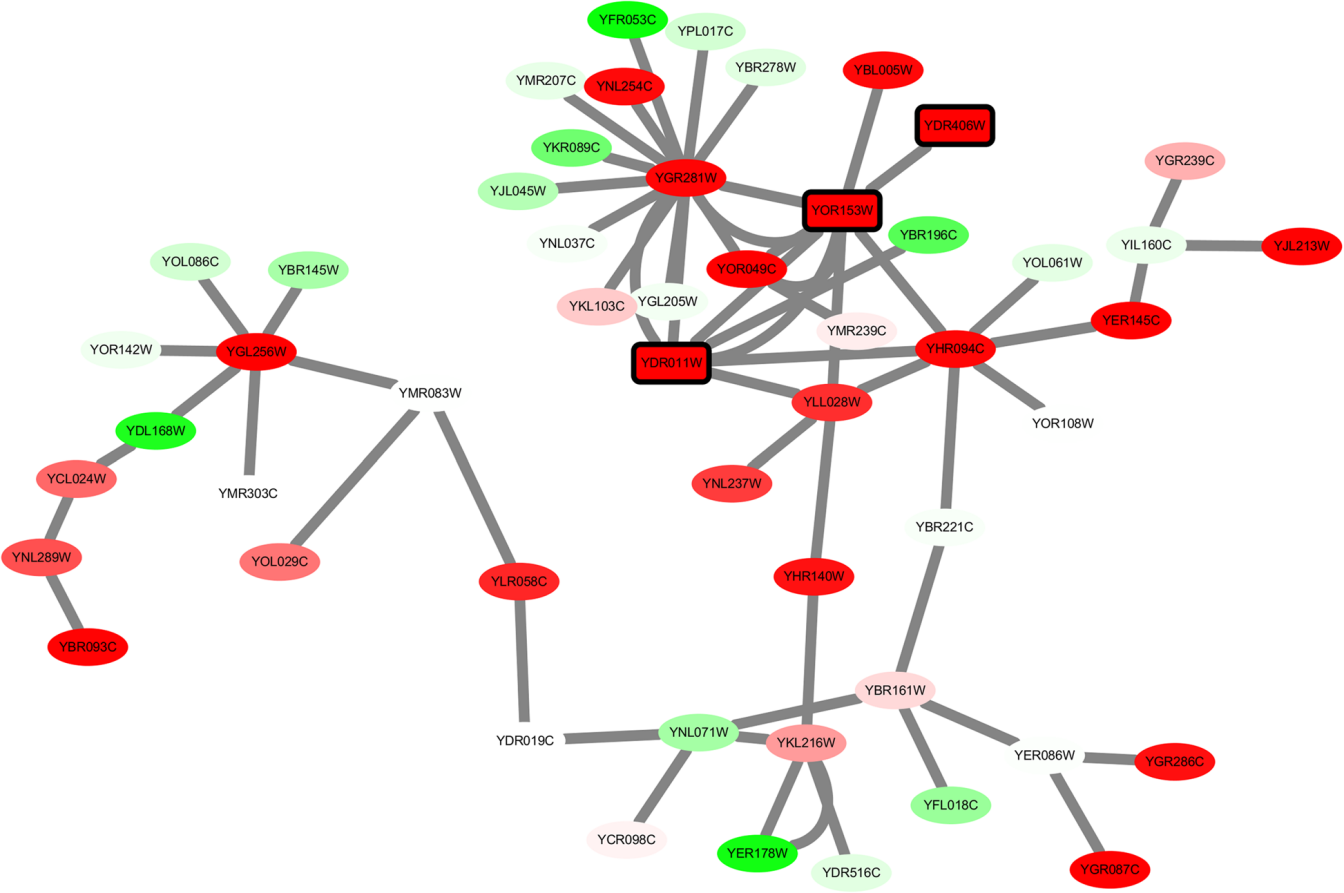
Visualization of module 3 identified by the proposed algorithm in network 2. Each node represents for a gene. The setting for node color is the same with Fig. 2. The turning point between red and green is set to the value *τ*=7.71×10^−6^. Three *rectangle shaped nodes* with *black border* are genes involved in drug export and are highly consistent in all modules. YDR406W is an ATP-binding cassette multidrug transporter. YDR011W is a ATP-binding cassette transporter. YOR153W is also an ATP-binding cassette multidrug transporter. The three genes serve as an important role in yeast’s resistance to diclofenac

The proposed algorithm is applied to network 2 with threshold *R*_*ratio*_=0.8 for KEGG pathway coverage score, resulting in a set of 12 Pareto solutions. Solutions on the Pareto front are chosen for gene ontology analysis on biological process. Surprisingly, all identified modules shows a high consistency in the annotation on drug reaction, which exactly reflects the cellular response for yeast under the diclofenac treatment. Three genes (YDR406W, YOR153W and YOR153W, all act as ATP-binding transporter, for detailed functional explanation, see caption in Fig. 6) that play an important role in the cellular reaction and resistance to drug treatment are discovered in all the 12 modules, indicating the accuracy and robustness of searching algorithm.

Similar to the analysis methods for results in network 1, 3 representative modules on Pareto front with different trade-off between active score *S*_*A*_ and pathway coverage score *R*_*A*_ are select for gene ontology annotation (see Table 4) and visualization (Fig. 6). From Table 4 we can see that as pathway score *R*_*A*_ increases, size of module increases and the annotation includes a larger range of biological processes. As drug reaction is considerably complicated response that involves a series of up or down regulation in related function groups such as protein kinase pathway, ribosome biogenesis, rRNA processing and zinc-responsive genes [32], the enriched annotation in modules with higher *R*_*A*_ provides a guidance of deciding which functional groups to look into as it combines both prior knowledge from existing interaction database and novel information from gene expression data specific for given experimental conditions.

**Table 4:** Gene ontology results of 3 modules on Pareto front detected by the proposed algorithm in network 2

Module	Size	*S*_*A*_	*R*_*A*_	Typical GO terms	*p*-value
1	34	91.01	0	Drug export	1.79×10^−03^
				Cellular response to drug	4.71×10^−02^
2	39	57.56	4	Drug export	2.84×10^−03^
3	62	46.332	8	Drug export	1.21×10^−02^
				Amino acid catabolic process to alcohol via Ehrlich pathway	8.65×10^−09^
				Ethanol metabolic process	3.71×10^−06^
				NADH oxidation	3.73×10^−03^
				Glycolytic process	4.34×10^−03^
				Fermentation	1.40×10^−02^
				Macromolecule metabolic process	2.51×10^−02^

## Conclusion

An integrated multi-objective approach has been proposed for active module identification. The algorithm is motivated by the idea that incorporating prior information into data-driven method would provide new insights into sophisticated biological processes. We also designed an constraint based on algebraic connectivity to ensure the connectedness of the identified active modules.

We first applied our algorithm on a small molecular interaction network, which identified a set of Pareto solutions that represents different trade-off between prior knowledge and novel information from data. Gene Ontology analysis results show that it successfully identifies modules with relevant and reasonable biological interpretations. The algorithm was applied to the second network, The approach is then applied on a microarray dataset from diclofenac-treated yeast cells and identify modules to elucidate the molecular mechanisms of diclofenac toxicity and resistance. The algorithm identifies accurate and consistent modules with biological function densely related to given cellular response, proving that the integrated approach for network construction is feasible and that the proposed algorithm is able to identify biologically meaningful modules in large scale network.

## Abbreviations

BUM: Beta-uniform mixture
FDR: False discovery rate
GEO: Gene expression omnibus
GO: Gene ontology
KEGG: Kyoto encyclopedia of genes and genomes
